# Development of a knee joint magnetic resonance imaging (MRI)-based model for finite element analysis (FEA) applications

**DOI:** 10.1051/sicotj/2026009

**Published:** 2026-04-20

**Authors:** Angelo V. Vasiliadis, Kalliopi Valsamidou, Alexandros Chortis, Dimitrios Chytas, George Noussios, George Paraskevas, Konstantinos Katakalos, Aikaterini Vassiou

**Affiliations:** 1 Department of Anatomy, Faculty of Medicine, University of Thessaly 41500 Larissa Greece; 2 Orthopaedic Surgery and Sports Medicine Department, FIFA Medical Center of Excellence, Croix-Rousse Hospital, Lyon University Hospital Lyon France; 3 Department of Physical Education and Sports Sciences at Serres, Aristotle University of Thessaloniki 62110 Agios Ioannis- Serres Greece; 4 Department of Architecture, Faculty of Engineering, Aristotle University of Thessaloniki 54124 Thessaloniki Greece; 5 Laboratory for Experimental Strength of Materials and Structures, Department of Civil Engineering, Faculty of Engineering, Aristotle University of Thessaloniki 54124 Thessaloniki Greece; 6 Basic Sciences Laboratory, Department of Physiotherapy, University of Peloponnese 23100 Sparta Greece; 7 School of Medicine, European University of Cyprus 2404 Nicosia Cyprus; 8 Department of Anatomy and Surgical Anatomy, School of Medicine, Faculty of Health Sciences, Aristotle University of Thessaloniki 54124 Thessaloniki Greece

**Keywords:** Knee joint biomechanics, Magnetic resonance imaging, Finite element analysis, Ligaments, Meniscus

## Abstract

*Introduction*: The knee is a biomechanically complex joint supported by multiple anatomical structures, making it vulnerable to multiple injuries. Finite element analysis is a valuable tool for studying joint biomechanics, particularly in pre-operative planning and injury evaluation. However, most models are based on computed tomography, which limits soft tissue visualization. Thus, a magnetic resonance imaging-based finite element model of the knee, incorporating bones, ligaments, tendons, cartilage, and menisci, was developed to improve realism and clinical relevance in biomechanical simulations. *Materials and methods*: Magnetic resonance imaging data were obtained from a healthy adult male using a 1.5T scanner and processed using RETOMO and Rhinoceros software for 3D reconstruction and modeling. Meshes were cleaned, optimized, and anatomically validated. All major knee structures were modeled, including the femur, tibia, fibula, patella, cruciate and collateral ligaments, patellofemoral ligaments, quadriceps and patellar tendons, menisci, and articular cartilage. *Results*: The resulting model reconstructed both hard and soft tissues of the knee joint with high anatomical fidelity, based on direct MRI segmentation and literature-supported anatomical definitions. The use of magnetic resonance imaging enabled high-resolution identification of soft tissues, while advanced mesh refinement preserved anatomical detail with optimized file management. The inclusion of structures like the anterolateral ligament and patellofemoral ligaments expands the model’s clinical relevance in addressing a wider range of knee pathologies. *Conclusion*: This magnetic resonance imaging-based finite element analysis model provides a detailed and comprehensive, representation of the healthy human knee, including bones, cartilage, menisci, and tendons. While some ligament attachment points were derived from literature rather than MRI data, the model provides a foundation for future biomechanical studies, surgical planning and personalized treatment simulations.

## Introduction

The knee is the largest and most complex joint in the human body. It plays a vital role in mobility and is vulnerable to a variety of injuries due to its intricate anatomy and the high mechanical demands placed upon it [1]. The primary components of the knee joint include the femur, tibia, fibula, and patella. These bones are supported by various soft tissues, such as joint capsule, menisci, ligaments, tendons, fat pads, and muscles, all of which work synergistically to provide structural and biomechanical stability [1, 2]. Several anatomical structures contribute to the knee stability, including the medial and lateral menisci and key ligaments, such as the anterior cruciate ligament (ACL), posterior cruciate ligament (PCL), medial collateral ligament (MCL), lateral collateral ligament (LCL), anterolateral ligament (ALL), medial patellofemoral ligament (MPFL), lateral patellofemoral ligament (LPFL). Additionally, tendons such as the quadriceps tendon (QT) and patellar tendon (PT) play important biomechanical roles [1, 3].

Magnetic resonance imaging (MRI) is a widely used imaging modality for detailed assessment of both bone and soft tissues. It is particularly valuable for detecting and characterizing traumatic and non-traumatic knee injuries, assisting in diagnosis, treatment planning, and monitoring [4]. With advances in computational technologies, finite element analysis (FEA) has emerged as a powerful tool for biomechanical modeling [1, 5]. FEA enables the creation of detailed three-dimensional (3D) anatomical models and allows simulation of complex loading conditions that are difficult to replicate in traditional experimental setups. As a non-invasive, repeatable, and precise approach, FEA supports the evaluation of stress, strain, displacement, and rotation, thereby aiding in injury mechanism analysis and operative planning [5, 6]. While many studies have focused on FEA models derived from computed tomography (CT) or MRI data [7–12], these models often prioritize bony structures and provide limited representation of the detailed soft tissue architecture of the knee joint.

Thus, the primary objective of the present study was to develop an anatomically comprehensive, MRI-based model of the knee joint, including the majority of the critical anatomical structures, and it is intended to serve as a valuable reference for future biomechanical studies and clinical applications.

## Materials and methods

### General information

A healthy male volunteer (32 years old, 179 cm, 74 kg, body mass index 23.1 kg/m^2^) with no history of knee pathology, trauma, or surgery participated in this study. Ethical approval was obtained from the university’s institutional review board, and written informed consent was provided by the volunteer.

### Acquisition of MRI data

An MRI scan was first performed to obtain the anatomical structures on a 1.5T system (MAGNETOM^®^ Avanto Tim + Dot system, Siemens, Munich, Germany) with an 8-channel knee coil. The MRI examination was performed with the knee in slight flexion with the STIR, T1, and T2 acquisitions in all three anatomical planes (sagittal, coronal, transverse). Acquisition parameters were as follows: FoV read, 150 mm; FoV phase, 100%; slice thickness, 0.6 mm; slice spacing, 0.6 mm; resolution, 512 × 512; TE, 4.76 ms; TR, 11 ms. A total of 1130 Digital Imaging and Communication in Medicine (DICOM)-format images were acquired.

### 3-dimensional (3D) reconstruction process

The DICOM (.dcm) images were imported into RETOMO (BETA CAE Systems International AG, Root, Switzerland). Initial segmentation was performed automatically and refined manually. Segmentation was based on grayscale intensity, and iterative seed/grow methods were used to enhance segmentation accuracy. The final 3D meshes were exported and saved in the stereolithography (STL) format.

### 3D knee modeling

The STL files were imported into Rhinoceros (Rhino & SR38, Robert McNeel and Associates for Windows, Washington DC, USA). While major osseous structures, menisci, and cartilage were directly segmented from MRI data, several ligamentous and tendinous structures were reconstructed using a hybrid approach that combined MRI visualization with established anatomical descriptions from the literature. This approach was adopted due to the limited visibility of fine ligament boundaries on conventional clinical MRI sequences. Anatomical attachment sites and fiber trajectories were defined based on published anatomical studies and subsequently adjusted to match subject-specific MRI morphology.

The initial geometry of the tibia and fibula, exported as a single conjoined mesh from the 3D reconstruction process, consists of approximately 528,000 triangles and was separated into individual bones within Rhinoceros. The femur consisted of 536,000 triangles, while the patella included 50,000. To optimize the model for further processing, the meshes were reduced to 10,000 triangles for the tibia and fibula, 5,000 for the femur, and 2,500 for the patella using the ReduceMesh Rhino function. This represents an approximate 98–99% reduction in polygon count achieved across all structures while maintaining anatomical accuracy. Additionally, the Mesh decimation significantly reduced the model’s size, enabling overall easier file management and smoother mesh manipulation. Rhinoceros software has been employed in similar biomedical workflows to refine 3D anatomical surfaces derived from medical imaging data prior to downstream analysis or fabrication [13, 14]. Rhinoceros allows accurate creation and manipulation of complex anatomical surfaces and free-form geometries, such as bone and soft tissue structures, making it suitable for reconstructing detailed patient-specific anatomy [13].

Peripheral noise and artifacts were removed to clean the external geometry of the model, followed by the elimination of internal bone artifacts generated during the 3D reconstruction process. All bone geometries were then converted into quad meshes using Rhino's QuadRemesh command, resulting in cleaner topology and the elimination of any intersecting faces. Any remaining surface holes were closed, and all face normals were verified and corrected when necessary to ensure geometric consistency.

Following initial cleanup, the ligament modeling was initiated by defining the anatomical attachment points between the relevant bones. Between each pair of attachment points, two to five guide curves were created to define the path, thickness, and shape of the ligament. The final ligament shapes were created using Rhino’s Sweep1 or Sweep2 commands. In cases requiring more complex forms, the Multipipe function was applied after inserting several cross-sections along the guide curves between the attachment point outlines. The same methodology was followed for modeling the tendons.

For the articular cartilage, most geometries were designed using a single top view by designing the cartilage footprint and then projecting it onto the respective bone. For the femoral articular cartilage, a multiplanar spatial curve was manually defined. Unlike the other cartilage structures, which were constructed in a single top view, the femoral outlines were designed and projected separately across bottom, front, and back views, and afterwards were joined into a single curve. The final curve continuously transitions across curved surfaces and varying anatomical planes, following the distal femur. Once the cartilage outlines were finalized, a 1 mm thick mesh was created by trimming a copy of a bone’s surface and then applying thickness to it using Weaverbird’s Mesh Thicken function to create the tissue’s volume.

Finally, the medial and lateral menisci were constructed by designing the characteristic C-shaped footprints of the medial meniscus (MM) and lateral meniscus (LM) on top of MTP and LTP cartilages. A series of inward polylines was then generated using Grasshopper’s Clipper Polyline offset function. These contour lines were distributed at varying heights to approximate the natural wedge-shaped cross-section of each meniscus. The final 3D geometry was obtained by patching the curves, resulting in anatomically realistic, crescent-shaped meniscal surface structures.

All anatomical structures were identified from the MRI images with additional reference to descriptions found in the literature [15–20] and confirmed by two experienced orthopaedic surgeons. The 3D modeling was performed by an experienced architect under the guidance and supervision of an orthopaedic surgeon. The finalized 3D knee model can be made available to other researchers upon reasonable request. All anatomical structures can be exported in standard 3D format, such as STL, enabling replication, modification, and use in other FEA studies.

## Results

**Figure 1:**
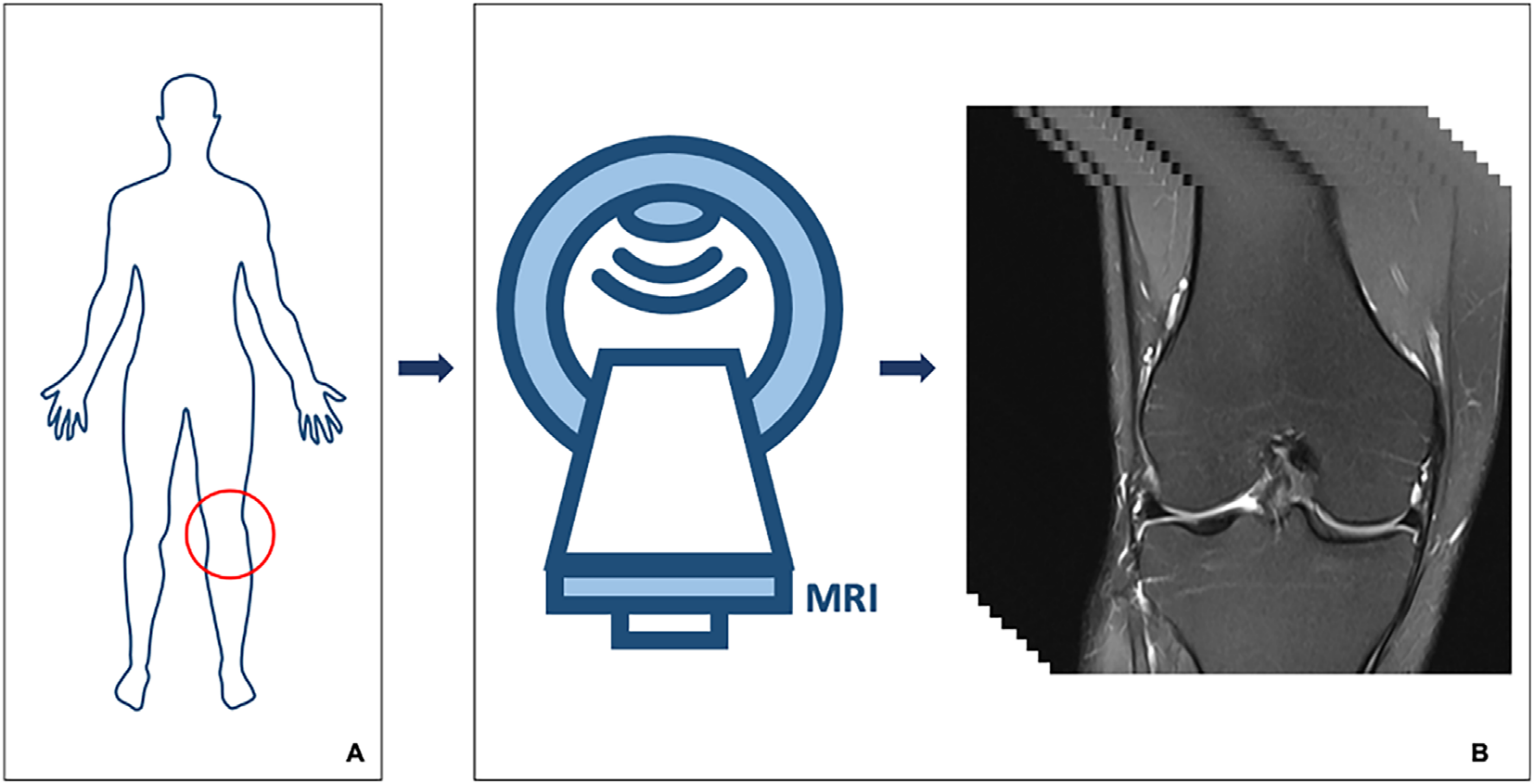
The left knee of a healthy individual (A) was imaged using magnetic resonance imaging (MRI) to obtain detailed anatomical views (B).

**Figure 2:**
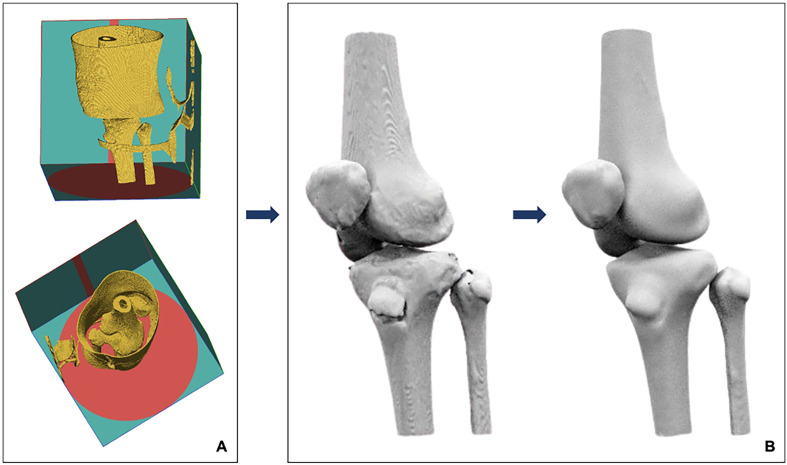
MRI data were imported into RETOMO, and the four knee joint bones were identified and incorporated into the 3D model (A). Post-processing of cleaned bone geometries following noise removal, artifact elimination, quadrilateral mesh conversion, and surface hole closure (B).

An MRI of the left knee (Figure 1) obtained from a healthy individual was used to develop an MRI-based FEA model. The imaging dataset demonstrated normal joint morphology, with intact osseous structures, cartilage surfaces, menisci, and ligaments. No pathological findings, anatomical variations, or degenerative changes were identified. The knee joint was imaged apprpximately10 cm proximal and distal to the joint line. All anatomical structures were either segmented directly from the patient’s MRI data or and reconstructed using literature-based anatomical definitions adapted to the subject-specific MRI geometry [15–20]. The MRI data were imported into RETOMO software, where all relevant bones were successfully identified. As expected, four bones forming the knee joint were clearly visualized and incorporated into the 3D model (Figure 2A). After removal of noises and artifacts, as well as the elimination of internal bone irregularities, the bone geometries were converted into quadrilateral meshes. Remaining surface holes were closed, resulting in a cleaner anatomical representation and improved geometry consistency (Figure 2B).

**Figure 3:**
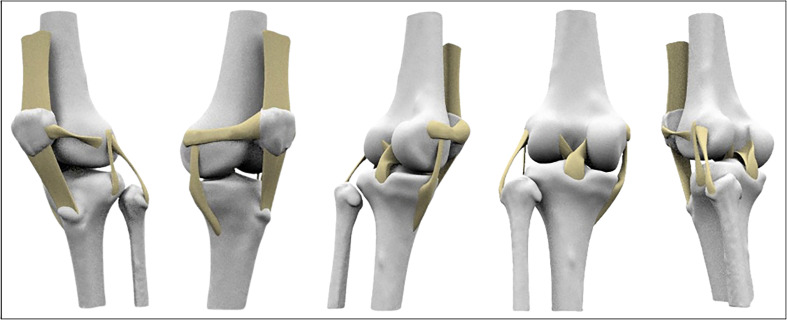
Post-processing ligament and tendon models were created by defining anatomical attachment points and generating geometries.

**Figure 4:**
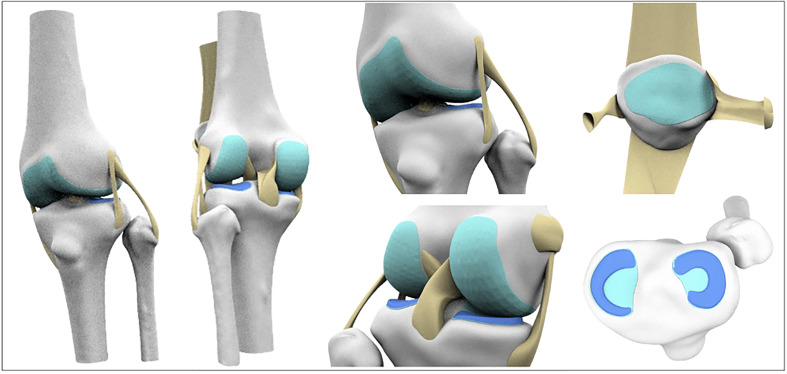
Reconstruction of articular cartilage and menisci geometries. Cartilage was modeled by projecting anatomical footprints onto bone surfaces, with the femoral cartilage requiring a multi-planar approach; menisci were generated from C-shaped footprints and height-varying contour lines to capture their wedge-shaped, crescent-like anatomy.

Ligaments (ACL, PCL, ALL, MCL, LCL, MPFL, LPFL) and tendons (quadricep and patellar) were modeled by defining anatomical attachment points, generating guide curves to capture their shape and thickness, and constructing final geometries using Rhino’s Sweep and multiple functions (Figure 3). Articular cartilage geometries were successfully reconstructed by projecting anatomically defined footprints onto bone surfaces, with the femoral cartilage requiring a multi-planar approach to accurately capture its complex curvature. The finalized cartilage structures were generated as 1 mm thick meshes, reflecting realistic anatomical shape and spatial continuity across joint surfaces. The medial and lateral menisci were accurately reconstructed as anatomically realistic, crescent-shaped 3D structures by offsetting their C-shaped footprints on the tibial cartilage and generating wedge-shaped cross-sections through height-varying contour lines (Figure 4). The reconstructed geometries were anatomically consistent with published descriptions of knee morphology, although no quantitative geometric validation against cadaveric or imaging ground truth was performed.

## Discussion

This study presents a detailed MRI-based knee joint model for FEA, including major and secondary stabilizers, with an emphasis on anatomical completeness rather than quantitative validation of geometric accuracy. MRI enables superior soft-tissue visualization and reconstruction compared with traditional methods, aiding understanding of joint function and injury mechanisms. It is important to note that although MRI provides excellent soft tissue contrast, not all ligamentous structures can be fully delineated with sufficient clarity for direct segmentation. Consequently, several ligaments were reconstructed using manually defined attachment points and trajectories informed by established anatomical literature. While this introduces a degree of uncertainty at the individual subject level, this methodology is commonly employed in computational knee modeling and enables the inclusion of clinically relevant stabilizing structures that are otherwise difficult to segment directly from MRI data.

The development of FEA models of the human joints has been widely described in the literature [7–9, 21]. These models are commonly used to analyze joint biomechanics under various physiological and pathological conditions. Watanabe et al. constructed a CT-based finite element model (FEM) of the knee joint to predict intra-articular stress and strain in the load response phase of walking, using gait analysis. Their work highlights the complex biomechanical behavior of the knee joint during the FEM analysis [7]. Interestingly, Kozaki et al. developed an FEA model from patient DICOM data obtained via CT to compare the effects of medial meniscus extrusion in relation to variations in knee alignment, ranging from varus to valgus [8]. The creation of an FEA model enables detailed biomechanical analysis, allowing for precise assessment of how anatomical variations influence joint stress. Moreover, Kang et al. [21] developed a subject-specific finite element knee model of a healthy lower extremity using 3D anatomical data from CT images, in order to evaluate forces exerted on cruciate ligaments and contact stresses on the tibiofemoral and patellofemoral joints in relation to three different posterolateral corner reconstruction techniques. They emphasized the advantages of computational models over in vitro studies in terms of methodological quality.

MRI-based FEA models have emerged as a powerful computational tool for knee biomechanics studies, but they also present some limitations and challenges. While CT-based FEA models are effective for visualizing and modeling bony structures, MRI-based FEA models offer superior visualization of soft tissues, including ligaments, tendons, cartilage, and menisci, which are crucial for understanding joint kinematics and pathology [1, 22]. However, the process of converting MRI data into a 3D FEA model is more complex and time-consuming, requiring significant manual intervention for segmentation and meshing [23]. Our model utilized MRI data to achieve a more comprehensive anatomical representation of the knee, allowing for more accurate biomechanical simulation of soft tissue behavior. This provides a significant advantage for assessing injuries, such as ligament/tendon tears, cartilage damage, or meniscal degeneration, and can be particularly valuable in pre-operative planning and post-operative evaluation [4, 24].

Furthermore, one of the major strengths of the present study is the inclusion of nearly all the major anatomical structures of the knee joint. Most existing models either focus on bone and cartilage or include only a limited number of ligaments, often excluding secondary stabilizers due to imaging and segmentation constraints [7–9, 21]. By including anatomical structures, such as ALL, as well as the MPFL and LPFL, this model enhances its capacity to assess a broader spectrum of clinical scenarios. These additions are particularly relevant in the area of knee surgery, where understanding the roles of secondary anatomical stabilizers is crucial for reconstructive surgeries, especially in rotational instability cases and patellofemoral disorders.

Interestingly, the future of MRI-based FEA models is promising. Advances in technology, particularly the integration of artificial intelligence (AI) and machine learning (ML), are expected to streamline the process of model creation [25]. These technologies could enable automated segmentation and meshing, significantly reducing the time and effort required to build a patient-specific model, making it more feasible for clinical use [1, 25]. Additionally, the development of more sophisticated models will lead to more realistic simulations, improving our understanding of joint biomechanics and pathologies like ligament tears [4, 24–26]. As computational power continues to increase, it will become possible to perform more detailed and complex analyses of the human body, such as simulating the effects of different surgical techniques or predicting the progression of knee diseases, like meniscal tears, over time [7, 23, 25, 26]. These will ultimately lead to better pre-operative planning, more personalized treatment strategies, and improved patient post-operative outcomes.

While the present study focused on developing a detailed MRI-based FEA model of the knee joint, the model provides a robust foundation for future clinical applications. Potential uses include simulation of ligament reconstruction techniques, such as ACL, PCL, and MPFL reconstruction, allowing evaluation of post-operative joint kinematics and ligament loading. The model can also support planning and optimization of meniscal repair techniques, root repair with bone tunnel, suture anchor, or all-inside approaches, as well as assessment of meniscal or cartilage injury mechanics under physiological or pathological loading conditions. Additionally, the model enables patient-specific biomechanical simulations that could inform pre-operative planning, predict surgical outcomes, and guide post-operative rehabilitation strategies. Although clinical case simulations were not performed in this study, the model is designed as a versatile platform for future research aimed at translating computational findings into clinical practice.

This study has several potential limitations. First, it was based on MRI data from a single healthy male subject and therefore does not capture gender-specific anatomical differences or inter-individual variability. While this approach was appropriate for developing a detailed MRI-based FEA knee model, future studies incorporating female subjects and a larger cohort are warranted to enhance generalizability and enable population-based analyses. Second, a 1.5T MRI system with updated software and an advanced protocol was used in the present study. Although 3T MRI scanners may offer certain advantages, MRI remains a reliable modality for detecting knee injuries. Moreover, a systematic review by Cheng and Zhao [27] demonstrated that both 1.5T and 3.0T MRI systems provide high diagnostic accuracy for meniscal and ligament evaluation, with no significant differences in diagnostic performance between them. Another limitation of the present model is that certain ligament attachment points were defined based on anatomical literature rather than being fully derived from subject-specific MRI data, which may reduce individual-level geometric precision but enhances anatomical completeness.

## Conclusion

An anatomically comprehensive MRI-based FEA model of the human knee was successfully developed, encompassing bones, articular cartilage, menisci, ligaments, and tendons. By leveraging high-resolution MRI data and advanced modeling tools, the present study addresses the limitations of previous CT-based models, especially in representing soft tissue anatomy. By including secondary stabilizers, such as the ALL and patellofemoral ligaments, the model offers broader clinical relevance, supporting simulations for ligament reconstruction, instability, and patellofemoral disorders. Future integration of AI and automated tools may streamline model generation, promoting patient-specific simulations and advancing personalized orthopaedic care.

## Data Availability

The data that support the findings of this study are available from the corresponding author upon reasonable request.
